# Supplementary material for: I am where I believe my body is: The interplay between body spatial prediction and body ownership
Source: PLoS One. 2024 Dec 12;19(12):e0314271. doi: 10.1371/journal.pone.0314271 (PMC11637335; doi:10.1371/journal.pone.0314271)
Supplement: S1 Appendix — (DOCX) [file pone.0314271.s001.docx]

**S1 Appendix. Embodiment Scale (ES)**

| Factor | Item (Factor) | From -3 to +3, how much do you agree with the following statements.  “During the block …” |  |  |  |  |  |  |  |
| --- | --- | --- | --- | --- | --- | --- | --- | --- | --- |
| Embodiment | 1 (E1) | ...it seemed like I was looking directly at my own hand/leg, rather than at a virtual hand/leg | -3 | -2 | -1 | -0 | +1 | +2 | +3 |
|  | 2 (E2) | ...it seemed like the virtual hand/leg began to resemble my real hand/leg | -3 | -2 | -1 | -0 | +1 | +2 | +3 |
|  | 3 (E3) | ...it seemed like the virtual hand/leg belonged to me | -3 | -2 | -1 | -0 | +1 | +2 | +3 |
|  | 4 (E4) | ...it seemed like the virtual hand/leg was my hand/leg | -3 | -2 | -1 | -0 | +1 | +2 | +3 |
|  | 5 (E5) | ...it seemed like the virtual hand/leg was part of my body | -3 | -2 | -1 | -0 | +1 | +2 | +3 |
|  | 6 (E6) | ...it seemed like my hand was in the location where the virtual hand/leg was | -3 | -2 | -1 | -0 | +1 | +2 | +3 |
|  | 7 (E7) | ...it seemed like the virtual hand/leg was in the location where my hand/leg was | -3 | -2 | -1 | -0 | +1 | +2 | +3 |
|  | 8 (E8) | ...it seemed like the touch I felt was caused by the paintbrush touching the virtual hand/leg | -3 | -2 | -1 | -0 | +1 | +2 | +3 |
|  | 9 (E9) | ...it seemed like I could have moved the virtual hand/leg if I had wanted | -3 | -2 | -1 | -0 | +1 | +2 | +3 |
|  | 10 (E10) | ...it seemed like I was in control of the virtual hand/leg | -3 | -2 | -1 | -0 | +1 | +2 | +3 |
| Disembodiment | 11 (D1) | ...it seemed like I was unable to move my hand/leg | -3 | -2 | -1 | -0 | +1 | +2 | +3 |
|  | 12 (D2) | ...it seemed like I couldn’t really tell where my hand/leg was | -3 | -2 | -1 | -0 | +1 | +2 | +3 |
|  | 13 (D3) | ...it seemed like my hand/leg had disappeared | -3 | -2 | -1 | -0 | +1 | +2 | +3 |
|  | 14 (D4) | ...it seemed like my hand/leg was out of my control | -3 | -2 | -1 | -0 | +1 | +2 | +3 |
|  | 15 (D5) | ...it seemed like my hand/leg was moving towards the virtual hand/leg | -3 | -2 | -1 | -0 | +1 | +2 | +3 |
|  | 16 (D6) | ...it seemed like the virtual hand/leg was moving towards my hand/leg | -3 | -2 | -1 | -0 | +1 | +2 | +3 |
| Physical Sensations | 17 (P1_r) | ...the touch of the paintbrush on my hand/leg was pleasant | -3 | -2 | -1 | -0 | +1 | +2 | +3 |
|  | 18 (P2) | ...I had the sensation of pins and needles in my hand/leg | -3 | -2 | -1 | -0 | +1 | +2 | +3 |

The table reports the questionnaire’s items used to assess embodiment and disembodiment feelings. Participants rated their agreement on 18 questions on a seven-point Likert scale (-3 to +3). Ten questions aimed to capture the embodiment experience (i.e., from E1 to E10), six focused on disembodiment (i.e., from D1 to D6), and two addressed physical sensations (i.e., P1_r and P2). Items E8 and P1_r were not evaluated during T0 assessment because participants did not experience any touch during the visual exposure. Item P1_r was reversed before analyses.
