# Supplementary material for: I am where I believe my body is: The interplay between body spatial prediction and body ownership
Source: PLoS One. 2024 Dec 12;19(12):e0314271. doi: 10.1371/journal.pone.0314271 (PMC11637335; doi:10.1371/journal.pone.0314271)

**S2 Appendix.** **Models and Posterior Predictive Check for each analysis performed.**

The file shows the model adopted for each analysis, specifying priors, family, chains and iterations.

**Bayesian Regression on Embodiment**

*Model*

brm_emb <- brm(
 Embodiment ~ Time * Location * Illusion +
 (1 + Time + Location + Illusion|| ID),
 data = db_total,
 family = skew_normal,
 prior = c( set_prior("normal(0,0.5)", class = "b"),

set_prior("normal(0,1)", class = "sd"),

set_prior("normal(0,0.5)", class = "Intercept", lb=-3, ub=3)),
 sample_prior = T,

chains = 4,
 iter = 8000,
 cores = 15,
 save_pars = save_pars(all=T))

*Posterior predictive check*pp_check(brm_emb, ndraws = 500)


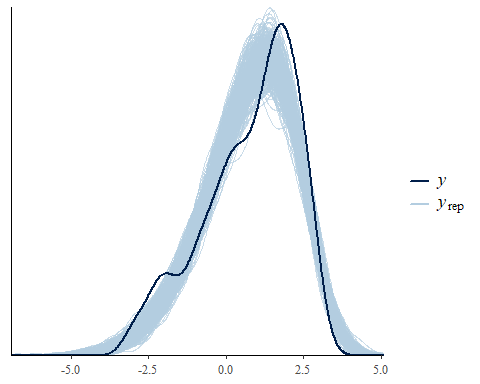


**Bayesian Regression on Disembodiment**

*Model*

brm_disemb <- brm(
 Disembodiment ~ Time * Location * Illusion +
 (1 + Time + Location + Illusion || ID),
 data = db_total,
 family = student,
 prior = c( set_prior("normal(0,0.5)", class = "b"),

set_prior("normal(0,1)", class = "sd"),

set_prior("normal(0,0.5)", class = "Intercept", lb=-3, ub=3)),

sample_prior = T,
 chains = 4,
 iter = 8000,
 cores = 15,
 save_pars = save_pars(all=T))

*Posterior predictive check*
pp_check(brm_disemb, ndraws = 500)


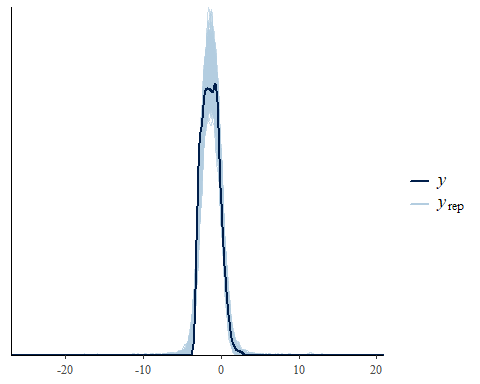


**Bayesian Regression on Physical Sensations**

The dependent variable distribution did not follow a Guassian shape but showed peaks around -3 and 0. Given that the cumulative distribution provided the most accurate fit for the data, we recode the variable. We transformed of the original scale from 1 to 7, thus creating an integer variable without zero. We used the default brm priors for this family distribution.

*Model*
brm_phys <- brm(
 PhysicR ~ Time * Location * Illusion +
 (1 + Time + Location + Illusion || ID),
 data = db_total,
 family = cumulative,

sample_prior = T,
 chains = 4,
 iter = 8000,
 cores = 15,
 save_pars = save_pars(all=T))

*Posterior predictive check*
pp_check(brm_phys, ndraws = 500)


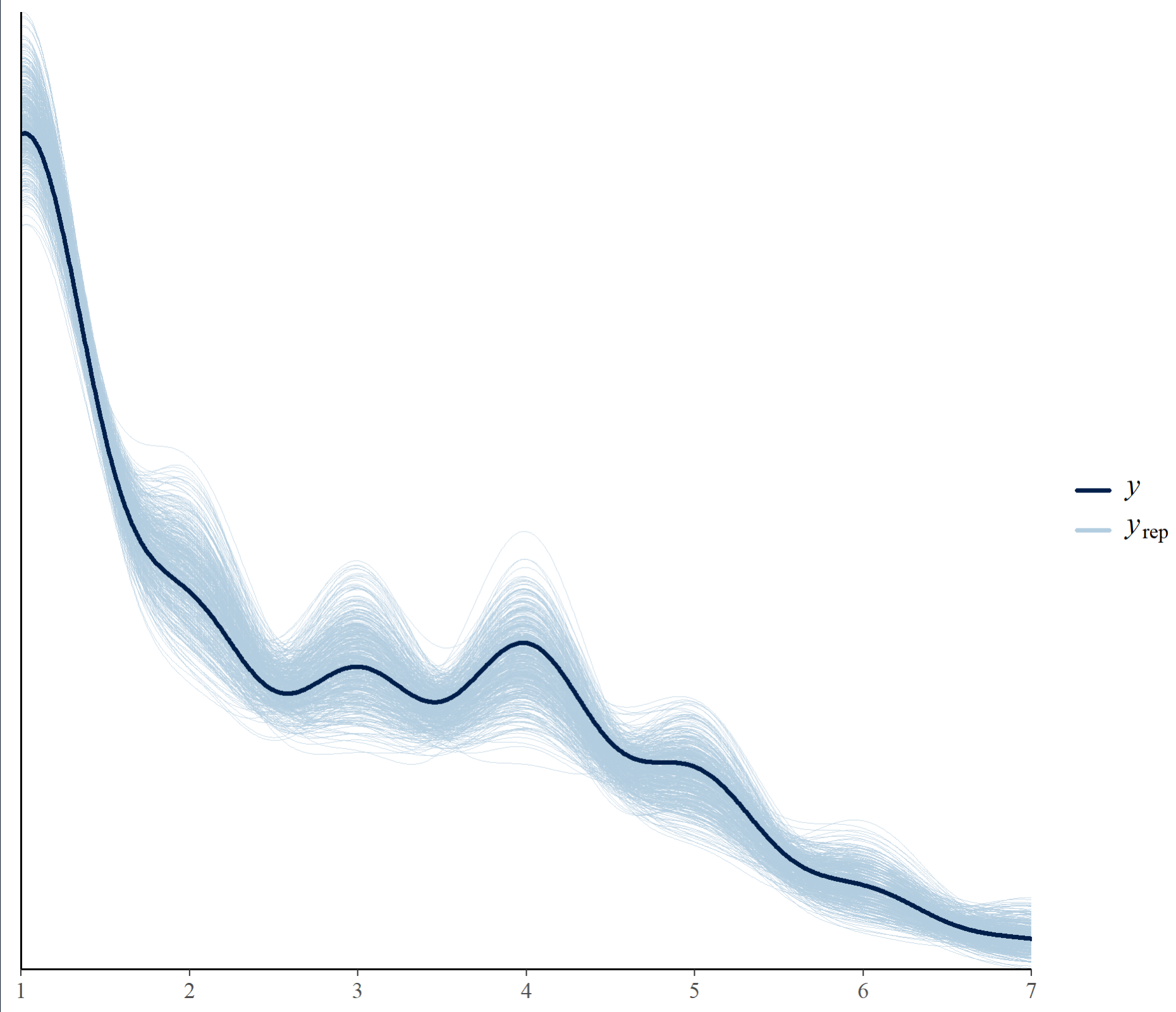


**Bayesian Regression on SCR-PP**

*Model*
brm_SCR <- brm(
 SCR.PP ~ Location * Illusion +
 (1 + Location + Illusion || ID),
 data = db_total,
 family = student,
 prior = c(set_prior("normal(0,0.5)", class = "b"),
 set_prior("normal(0,1)", class = "sd"),
 set_prior("normal(0,0.5)", class = "Intercept")),

sample_prior = T,
 chains = 4,
 iter = 8000,
 cores = 15,
 save_pars = save_pars(all=T),
 control = list(adapt_delta = 0.9))

*Posterior predictive check*
pp_check(brm_SCR, ndraws = 500)


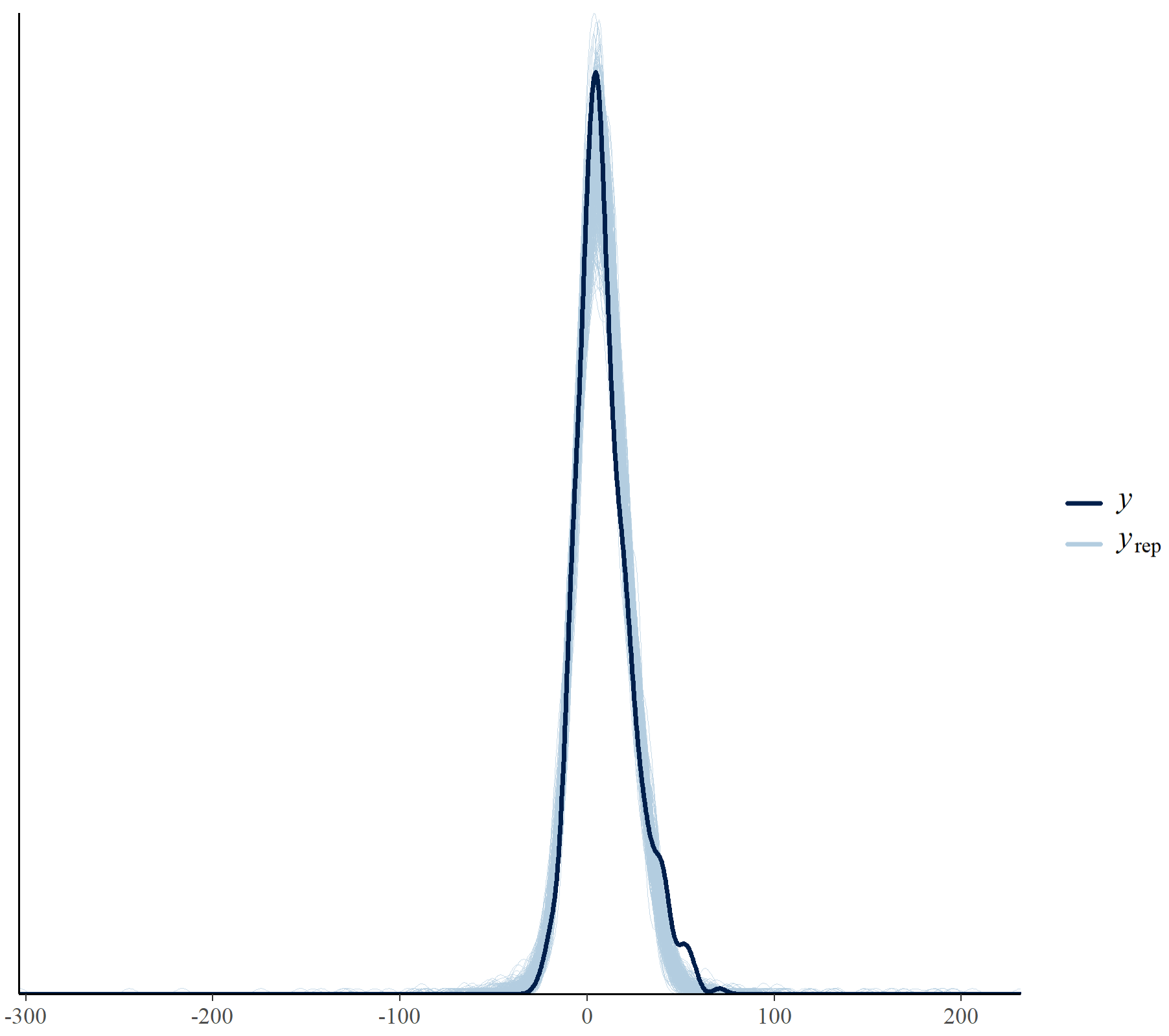


**Bayesian Regression on Proprioceptive Drift**

*Model*
brm_propD_trial <- brm(
 Prop.drift ~ Time * Location * Illusion +
 (1 + Time + Location + Illusion || ID + Trial),
 data = dt_propdrift,
 family = student,
 prior = c(set_prior("normal(0,0.5)", class = "b"),
 set_prior("normal(0,1)", class = "sd"),
 set_prior("normal(0,0.5)", class = "Intercept")),
 sample_prior = T,
 chains = 4,
 iter = 8000,
 cores = 15,
 save_pars = save_pars(all=T))

*Posterior predictive check*
pp_check(brm_propD_trial, ndraws = 500)


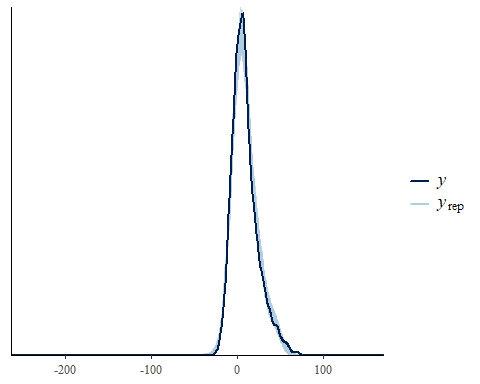


**Additional Analysis**

**Bayesian Regression on Question 6 in the vRHI, considering Order of Sessions (Group)**

The dependent variable distribution did not follow a Guassian shape. Given that the cumulative distribution provided the most accurate fit for the data, we recode the variable. We transformed of the original scale from 1 to 7, thus creating an integer variable without zero. We used the default brm priors for this family distribution.

*Model*

brm_Q6 <- brm(
 Q6r ~ Time * Location * Group +
 (1 + Time + Location || ID),
 data = db_arm,
 family = cumulative,
 chains = 4,
 iter = 8000,
 cores = 15)

*Posterior predictive check*
pp_check(brm_Q6, ndraws = 500)


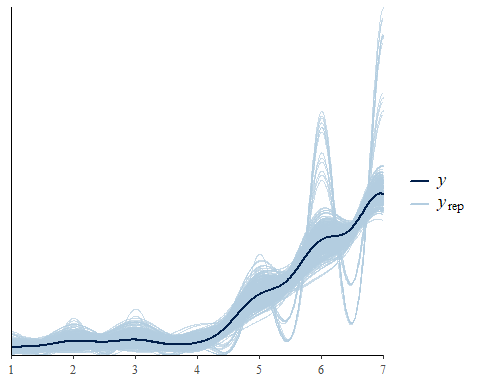


**Bayesian Regression on Proprioceptive Drift in vRHI, considering Order of Sessions (Group)**

*Model*

brm_pd <- brm(
 Prop.drift.M ~ Time * Location * Group +
 (1 + Time + Location || ID),
 data = db_arm,
 family = student,
 prior = c(set_prior("normal(0,0.5)", class = "b"),

set_prior("normal(0,1)", class = "sd"),
 set_prior("normal(0,0.5)", class = "Intercept")),

chains = 4,
 iter = 8000,
 cores = 15)

*Posterior predictive check*
pp_check(brm_pd, ndraws = 500)


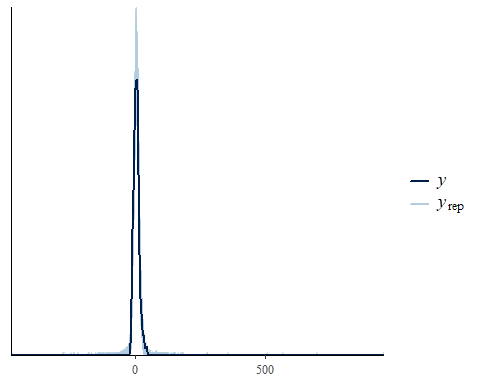


**Bayesian Regression on Question 6 in 1pp-FBI, considering Order of Sessions (Group)**

The dependent variable distribution did not follow a Guassian shape. Given that the cumulative distribution provided the most accurate fit for the data, we recode the variable. We transformed of the original scale from 1 to 7, thus creating an integer variable without zero. We used the default brm priors for this family distribution.

*Model*
brm_Q6.fbi <- brm(
 Q6r ~ Time * Location * Group +
 (1 + Time + Location || ID),
 data = db_FBI,
 family = cumulative,
 chains = 4,
 iter = 8000,
 cores = 7)

*Posterior predictive check*
pp_check(brm_Q6.fbi, ndraws = 500)


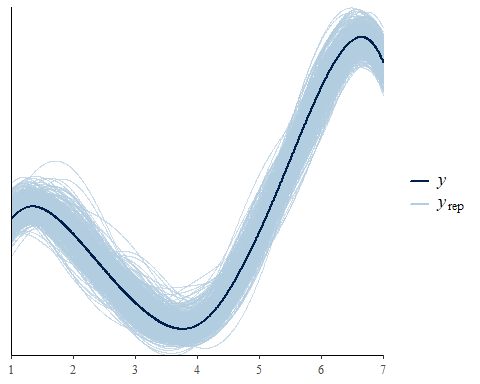

Supplement: S2 Appendix — (DOCX) [file pone.0314271.s002.docx]
