# Supplementary material for: I am where I believe my body is: The interplay between body spatial prediction and body ownership
Source: PLoS One. 2024 Dec 12;19(12):e0314271. doi: 10.1371/journal.pone.0314271 (PMC11637335; doi:10.1371/journal.pone.0314271)
Supplement: S3 Appendix — (DOCX) [file pone.0314271.s003.docx]

**S3 Appendix. Additional embodiment scale results.**

**Results of the Bayesian regression on Physical Sensations.** The table shows the mean (Estimate) and the standard deviation (Est.Error) of the posterior distribution of each effect with the 95% Credible Intervals (lower 95% CI, upper 95% CI). In bold, the posterior distributions without a zero overlapping.

| vRHI: Proprioceptive Drift | Estimate | Est. Error | Lower  95% CI | Upper  95% CI |
| --- | --- | --- | --- | --- |
| *Intercept[1]* | **-0.99** | **0.36** | **-1.71** | **-0.31** |
| *Intercept[2]* | **0.86** | **0.36** | **0.16** | **1.57** |
| *Intercept[3]* | **2.35** | **0.37** | **1.64** | **3.09** |
| *Intercept[4]* | **4.17** | **0.41** | **3.40** | **4.99** |
| *Intercept[5]* | **5.98** | **0.47** | **5.09** | **6.94** |
| *Intercept[6]* | **7.78** | **0.60** | **6.66** | **8.98** |
| *Time_T1_* | **2.78** | **0.43** | **1.98** | **3.65** |
| *Location_Misaligned_* | **0.47** | **0.19** | **0.09** | **0.84** |
| *Illusion_1ppFBI_* | **-0.57** | **0.24** | **-1.05** | **-0.10** |
| *Time_T1_*Location_Misaligned_* | -0.71 | 0.37 | -1.43 | 0.01 |
| *Time _T1_*Illusion_1ppFBI_* | -0.08 | 0.37 | -0.79 | 0.64 |
| *Location_Misaligned_*Illusion_1ppFBI_* | 0.38 | 0.36 | -0.34 | 1.09 |
| *Time_T1_*Location_Misaligned_*Illusion_1ppFBI_* | -0.30 | 0.72 | -1.73 | 1.11 |
