# Supplementary material for: I am where I believe my body is: The interplay between body spatial prediction and body ownership
Source: PLoS One. 2024 Dec 12;19(12):e0314271. doi: 10.1371/journal.pone.0314271 (PMC11637335; doi:10.1371/journal.pone.0314271)
Supplement: S4 Appendix — (DOCX) [file pone.0314271.s004.docx]

**S4 Appendix. SCR-PP results**

| vRHI: Proprioceptive Drift | Estimate | Est. Error | Lower  95% CI | Upper  95% CI |
| --- | --- | --- | --- | --- |
| *Intercept* | 1.23 | 0.11 | 1.02 | 1.44 |
| *Location_Misaligned_* | -0.04 | 0.06 | -0.16 | 0.08 |
| *Illusion_1pp-FBI_* | **-0.20** | **0.08** | **-0.37** | **-0.04** |
| *Location_Misaligned_*Illusion_1pp-FBI_* | -0.01 | 0.11 | -0.23 | 0.21 |

The table shows the mean (Estimate) and the standard deviation (Est.Error) of the posterior distribution of each effect with the 95% Credible Intervals (lower 95% CI, upper 95% CI). In bold, the posterior distributions without a zero overlapping.

The figure shows the results of the Bayesian regression on the SCR-PP, depending on Illusion and Location; Error bars indicate 95% Credible Interval limits. Results suggests that the SCR P-P is lower in the 1pp-FBI than in the vRHI.

**
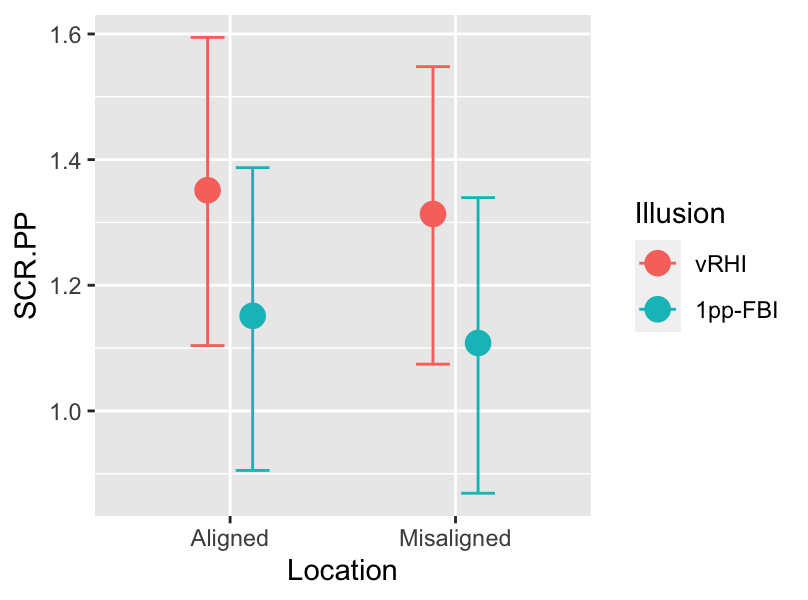
**
