# Supplementary material for: I am where I believe my body is: The interplay between body spatial prediction and body ownership
Source: PLoS One. 2024 Dec 12;19(12):e0314271. doi: 10.1371/journal.pone.0314271 (PMC11637335; doi:10.1371/journal.pone.0314271)
Supplement: S5 Appendix — (DOCX) [file pone.0314271.s005.docx]

**S5 Appendix. Additional analyses**

**Results of the 2 (Time) x 2 (Location) x2 (Order of Sessions) regression analysis on**

**Question 6 in the vRHI**

| vRHI: Question 6 (Embodiment Scale) | Estimate | Est. Error | Lower  95% CI | Upper  95% CI |
| --- | --- | --- | --- | --- |
| *Intercept[1]* | **-8.03** | **0.96** | **-10.12** | **-6.35** |
| *Intercept[2]* | **-6.21** | **0.78** | **-7.91** | **-4.86** |
| *Intercept[3]* | **-4.98** | **0.66** | **-6.40** | **-3.82** |
| *Intercept[4]* | **-4.34** | **0.61** | **-5.64** | **-3.26** |
| *Intercept[5]* | **-2.10** | **0.46** | **-3.06** | **-1.26** |
| *Intercept[6]* | **1.00** | **0.42** | **0.22** | **1.84** |
| *Time_T1_* | **1.09** | **0.32** | **0.52** | **1.75** |
| *Location_Misaligned_* | **-2.28** | **0.53** | **-3.41** | **-1.31** |
| *Order of Sessions_Misaligned_first_* | 0.64 | 0.77 | -0.86 | 2.17 |
| *Time_t1_*Location_Misaligned_* | 0.77 | 0.53 | -0.27 | 1.82 |
| *Time _T1_* Order of Session_Misaligned_first_* | 0.06 | 0.57 | -1.05 | 1.20 |
| *Location_Misaligned_ * Order of Session_Misaligned_first_* | **4.32** | **1.03** | **2.41** | **6.46** |
| *Time_T1_*Location_Misaligned_* Order of Sessions_Misaligned_first_* | 0.28 | 1.04 | -1.73 | 2.34 |

The table shows the mean (Estimate) and the standard deviation (Est.Error) of the posterior distribution of each effect with the 95% Credible Intervals (lower 95% CI, upper 95% CI). In bold, the posterior distributions without a zero overlapping. Given that the cumulative distribution provided the most accurate fit for the data, we recode the variable. We transformed of the original scale from 1 to 7, thus creating an integer variable without zero. As a consequence of the cumulative distribution, the model computed six intercepts.

**Results of the 2 (Time) x 2 (Location) x2 (Order of Sessions) regression analysis on**

**Question 6 in the 1pp-FBI**

| 1pp-FBI: Question 6 (Embodiment Scale) | Estimate | Est. Error | Lower  95% CI | Upper  95% CI |
| --- | --- | --- | --- | --- |
| *Intercept[1]* | **-6.07** | **0.92** | **-8.16** | **-4.56** |
| *Intercept[2]* | **-3.77** | **0.67** | **-5.28** | **-2.64** |
| *Intercept[3]* | **-2.67** | **0.57** | **-3.92** | **-1.67** |
| *Intercept[4]* | **-2.19** | **0.54** | **-3.36** | **-1.25** |
| *Intercept[5]* | -0.33 | 0.45 | -1.25 | 0.54 |
| *Intercept[6]* | **3.52** | **0.67** | **2.40** | **5.00** |
| *Time_T1_* | **2.43** | **0.50** | **1.60** | **3.57** |
| *Location_Misaligned_* | -7.91 | 1.29 | **-10.77** | **-5.79** |
| *Order of Sessions_Misaligned_first_* | 0.74 | 0.80 | -0.78 | 2.37 |
| *Time_t1_*Location_Misaligned_* | **2.69** | **0.64** | **1.48** | **4.01** |
| *Time _T1_* Order of Session_Misaligned_first_* | 0.86 | 0.70 | -0.43 | 2.34 |
| *Location_Misaligned_ * Order of Session_Misaligned_first_* | 0.24 | 1.41 | -2.53 | 3.05 |
| *Time_T1_*Location_Misaligned_* Order of Sessions_Misaligned_first_* | 0.62 | 1.15 | -1.64 | 2.89 |

The table shows the mean (Estimate) and the standard deviation (Est.Error) of the posterior distribution of each effect with the 95% Credible Intervals (lower 95% CI, upper 95% CI). In bold, the posterior distributions without a zero overlapping. Given that the cumulative distribution provided the most accurate fit for the data, we recode the variable. We transformed of the original scale from 1 to 7, thus creating an integer variable without zero. As a consequence of the cumulative distribution, the model computed six intercepts.

**Results of the 2 (Time) x 2 (Location) x2 (Order of Sessions) regression analysis on**

**Proprioceptive Drift in the vRHI**

| vRHI: Proprioceptive Drift | Estimate | Est. Error | Lower  95% CI | Upper  95% CI |  |  |
| --- | --- | --- | --- | --- | --- | --- |
| *Intercept* | **1.22** | **0.43** | **0.37** | **2.09** |  |  |
| *Time_T1_* | -0.12 | 0.31 | -0.72 | 0.48 |  |  |
| *Location_Misaligned_* | **-2.67** | **0.47** | **-3.57** | **-1.74** |  |  |
| *Order of Sessions_Misaligned_first_* | -0.19 | 0.47 | -1.11 | 0.73 |  |  |
| *Time_t1_*Location_Misaligned_* | **-0.87** | **0.43** | **-1.70** | **-0.02** |  |  |
| *Time _T1_* Order of Session_Misaligned_first_* | -0.19 | 0.42 | -1.02 | 0.63 |  |  |
| *Location_Misaligned_ * Order of Session_Misaligned_first_* | 0.18 | 0.47 | -0.74 | 1.10 |  |  |
| *Time_T1_*Location_Misaligned_* Order of Sessions_Misaligned_first_* | -0.20 | 0.47 | -1.14 | 0.73 |  |  |

The table shows the mean (Estimate) and the standard deviation (Est.Error) of the posterior distribution of each effect with the 95% Credible Intervals (lower 95% CI, upper 95% CI). In bold, the posterior distributions without a zero overlapping.
